# Protective Properties of Attenuated Strains of African Swine Fever Virus Belonging to Seroimmunotypes I–VIII

**DOI:** 10.3390/pathogens9040274

**Published:** 2020-04-09

**Authors:** Alexey D. Sereda, Vladimir M. Balyshev, Anna S. Kazakova, Almaz R. Imatdinov, Denis V. Kolbasov

**Affiliations:** Federal Research Center for Virology and Microbiology (FRCVM), 601125 Volginsky, Petushki area, Vladimir region, Russia; balyshevvm@rambler.ru (V.M.B.); almazlcf@yandex.ru (A.R.I.); kolbasovdenis@gmail.com (D.V.K.)

**Keywords:** African swine fever, attenuated strains, vaccines, seroimmunotypes

## Abstract

This article summarizes the study results on the generation of attenuated strains of African swine fever virus (ASFV) of seroimmunotypes I–VIII and the creation of live vaccines for temporary protection of pigs during a period of epizootics in the surveillance zone (a zone adjacent to the area of outbreak). These studies were initiated at the Federal Research Center for Virology and Microbiology (FRCVM, formerly VNIIVViM) at the time of introduction of the pathogen to the Iberian Peninsula in the middle of the 20th century. The developed experimental vaccines against ASFV seroimmunotypes I–V provided protection against virulent strains of homologous seroimmunotypes by day 14 after vaccination, lasting at least four months.

## 1. Introduction

African swine fever (ASF) is a highly contagious hemorrhagic disease of pigs caused by a large, cytoplasmic, icosahedral DNA virus (ASFV) with a genome size of 170–193 kbp. Virulent isolates kill domestic pigs within 7–10 days of infection. In chronic cases, ASF causes respiratory disorders and, in some cases, swelling around the leg joints and skin lesions. Domestic pigs can survive infection with less virulent isolates and, in doing so, it is possible to gain immunity to subsequent challenges with related virulent viruses [1,2,3,4,5,6,7,8].

No efficient vaccines against ASF have been developed, and inactivated-vaccine technologies have proven to be ineffective [9,10]. In the early 1960s, the prospects of development for live vaccines seemed encouraging [11]. During ASF epizootics in 1962–1963, 550,000 pigs in Portugal and 18,000 pigs in Spain were vaccinated with a live vaccine derived from an attenuated strain of the ASFV. A few months after vaccination, the number of sites affected by ASF in both countries increased by three to six times, with a death rate of 10%–50% of the vaccinated livestock, and the manifestation of clinical signs of the disease for a long period of time after vaccination [12].

From the 1970s to the 1990s, studies were conducted in the Federal Research Center for Virology and Microbiology (FRCVM, formerly VNIIVViM) on the selection of attenuated strains for the development of live vaccines against ASF [13].

According to the adopted concept, live vaccines were being developed for the temporary protection of vaccinated pigs at large pig-breeding complexes for the subsequent planned slaughter of pigs and their processing for cooked meat products. By their properties, attenuated strains had to be weakly or moderately reactogenic, not reversible, cause viremia limited in level and time, not cause autoimmune complications, and not be transmitted to naïve pigs when kept together with the vaccinated pigs. Vaccine preparations made on this basis had to be harmless to pigs at a 5–10-fold vaccination dose, and provide protection by 7–14 days in at least 75% of pigs, lasting four or more months. There were three main requirements for the use of such vaccines: (1) the restriction of the use of vaccine preparation to the first threatened zone, (2) it was only to be used in large pig farms, and (3) the slaughter of all vaccinated pigs processed for cooked sausages and stews must occur in the first four months, with strict observance of other veterinary and sanitary measures in accordance with the instructions for combating ASF [13].

## 2. Seroimmunotype Classification of ASFV Strains, Isolates, and Variants

In those and subsequent years, in parallel with studies on the biological properties of new isolates entering the institute, new data were accumulated on the seroimmunotypic plurality of ASFV. On the basis of serological typing by the hemadsorption inhibition assay (HADIA) [2] in combination with an immunobiological test (protection of pigs against fatal infection by a virulent test virus after immunization with an attenuated virus strain of the homologous serotype), the seroimmunotypical classification of ASFV was established in FRCVM (formerly VNIIVViM) [14,15,16]. Analysis of more than 100 virulent and attenuated strains provided the basis for this ASFV seroimmunological classification (Table 1). The strains assigned to the same group using the results of an immunobiological test and HADIA were combined into nine separate seroimmunotypes with respective reference strains: I—Lisbon-57 (L-57), II—Congo-49 (C-49), III—Mozambique-78 (M-78), IV—France-32 (F-32), V—TSP-80, VI—TS-7, VII—Uganda, VIII—Rhodesia and Stavropol 01/08 (including other strains of genotype II currently circulating in Eastern Europe), and IX—Davis [16,17,18]. Group X includes isolates of which the serotypic affiliation, according to HADIA data, does not correspond to the results of the immunobiological test. Therefore, isolates of Portugal-60 (P-60) and Spain-70 belong to Serotype IV but cause the death of immunized pigs that survived after a challenge with virulent strain France-32. Group XI includes the heterogeneous isolate of ASFV Kiravira-67 isolated in Tanzania. When it was passaged in various cell cultures and/or on immune pigs by contact, ASFV variants belonging to seroimmunotypes I and III were identified. From isolate Kiravira-67, strains TSP-80 and TS-7 were also obtained, which were different from ASF viruses belonging to seroimmunotypes I–IV. These strains were adopted as the reference strains of seroimmunotypes V (TSP-80) and VI (TS-7) [15,16,17].

This publication summarizes the main results of studies performed by the FRCVM (formerly VNIIVViM) for the production of attenuated ASFV strains belonging to various seroimmunotypes, and the development of live vaccines based on them.

### 2.1. Seroimmunotype I

By selective passages in primary cultures of pig bone marrow cells (PBMCs) and leukocytes of swine (LSs) of reference strain Lisbon-57 of the ASFV (seroimmunotype I), attenuated strain LK-111 was obtained. On day 14, after the inoculation of strain LK-111 to pigs at a dose of 10^7.0^ 50% hemadsorbing units (HAU_50_), protection against a subsequent intramuscular challenge with virulent strain Lisbon-57 at a dose of 10^4^ HAU_50_ was formed in only 50%–70% of the immunized pigs. Because of the low protection, prolonged viremia after inoculation to pigs, and autoimmune complications, strain LK-111 was not recommended for the development of vaccine preparations [19].

Virulent hemadsorbing strain Katanga-105 isolated in Zaire (DRC) caused the death of 87% of pigs (63% on days 7–13; 24%—by day 30), and the surviving pigs formed a resistance against reference strain Lisbon-57. To attenuate the Katanga-105 strain, the virus was passed in the PBMC culture by limiting virus dilutions with the selection of clones with low hemadsorbing activity. The selected virus variant, Katanga-115, only showed hemadsorption activity at a titer of 10^7.0^ 50% Tissue Culture Infectious Dose (TCID_50_) per mL of stock (TCID_50_/mL) and caused the death of 50% of pigs at infection doses of 10^6.0^–10^7.0^ TCID_50_. Then, nonhemadsorbing virus variant Kc-139 was obtained, which caused the death of 30% of the pigs, and the avirulent variant of Kc-149, 14 days after inoculation at a dose of 10^7.0^ TCID_50_, formed resistance in pigs to intramuscular infection with strain Lisbon-57 at a dose of 10^4.0^ HAU_50_. Subsequent nonhemadsorbing virus variant Katanga-160 (Kc-160), when administered intramuscularly to pigs at a dose of 10^7.5^ TCID_50_, caused a weak or moderate clinical response in 75%–80% of the pigs. Viremia lasted no more than 28 days. On days 5 and 15 after administration, the nonhemadsorbing virus was detected in pig blood in titers from 10^1.5^ to 10^2.5^ TCID_50_/mL and from 10^1.3^ to 10^2.0^ TCID_50_/mL, respectively. On day 14, 80%–100% of the pigs formed resistance to intramuscular infection of 10^4.0^ HAU_50_ of strain Lisbon-57. 

Fifteen days after vaccinated pigs were infected with Lisbon-57, the virus was detected in the blood in titers from 103.0 to 103.5 HAU50/mL, and after 30 days, the virus was not detected in the blood of most of the pigs. The pigs remained clinically healthy for four months, which was the observation period. Further study of the biological properties of nonhemadsorbing variant Kc-160 showed that it was stable after 30 passages in the cell culture of the PBMCs and did not revert after three consecutive passages in the pigs. Accordingly, attenuated nonhemadsorbing variant Kc-160 of ASFV of seroimmunotype I corresponded to the requirements for vaccines.

To assess the effects of using the Kc-160 variant on pigs with a lowered immune status, a group of 25 pigs with a low level of white blood cells and with symptoms of gastroenteritis, bronchopneumonia, and arthritis was formed. After intramuscular inoculation of the Kc-160 variant at a dose of 106.5 TCID50, 20% of these pigs died on days 9–14. At the autopsy, characteristic pathological changes to the acute form of ASF were not found. A nonhemadsorbing virus was isolated from the blood of these pigs in titers of 104.0–104.5 TCID50/mL.

As a result of passaging in the PBMC culture of virulent ASFV strain Katanga-78 of seroimmunotype I, attenuated hemadsorbing strain Katanga-350 was obtained. After vaccination of pigs with an experimental series of the vaccine made on the basis of Katanga-350, only 42% of the pigs had a slight temperature reaction. Blood viremia was 102.7 HAU50/mL on day 7 and 100.7 HAU50/mL on day 14. On days 20–30, the virus was not isolated from the blood. Transmission of the attenuated strain by contact from animal to animal, as well as reversion during five consecutive passages in pigs, was not established. Seven days after the inoculation of pigs with Katanga-350 at a dose of 107.0 HAU50, 50% of the pigs were resistant to infection with the Lisbon-57 strain at a dose of 10^4.0^ HAU_50_, and 80% of the pigs were resistant after 14, 21, 60, and 180 days. Katanga-350 met the requirements for vaccines and was considered as the main candidate for the production of protective preparations against ASFV seroimmunotype I [20].

### 2.2. Seroimmunotype II

As a result of a prolonged passage by limiting dilutions in the PBMC culture of virulent ASFV strain Congo-49 of seroimmunotype II, attenuated strain KK-202 was obtained. KK-202 had pronounced reactogenicity in pigs, caused depression, high temperature reaction (fever), and hemorrhages in parenchymal organs, and reactivated after inoculation in pigs that were immune against ASFV seroimmunotype IV. A 75% protection of pigs vaccinated with a dose of 104.0 HAU50 formed only after 21–28 days. Therefore, according to the immunobiological properties, ASFV strain KK-202 did not meet the vaccine-preparation requirements and was not further investigated.

Next, strain KK-202 was passaged by the method of limiting dilutions and selective sorption on sensitive cells. Attenuated hemadsorbing strain KK-262/C, obtained as a result of selective passages, did not revert during five consequent passages in pigs, did not reactivate after inoculation to pigs that were resistant to ASFV of seroimmunotype IV, and was not transmitted by contact when vaccinated and naïve pigs were kept together. When this strain was administered intramuscularly, it was characterized as weakly reactogenic (in individual pigs, vaccinated at doses of 106.0–107.5 HAU50, body temperature increased to 40.6–40.9 °C for 3–5 days). Protection against challenge with homologous virulent reference strain Congo-49 at a dose of 104.0 HAU50 was formed in 75%–85% of pigs on day 14 after vaccination and lasted for a period of at least four months. On days 7–14, the titer of the virus in the blood of pigs after inoculation with strain KK-262/C was in the range of 102.0–103.5 HAU50/mL. Vaccination of pigs with two doses (administered on a seven-day interval) of the 25× concentrated (PEG 6000) preparation of this virus induced protection on day 14 in 80%–100% of the pigs. The main biological properties of strain KK-262/C were maintained for 20 passages of cultivation in cell culture PPK-66b or PBMCs. Therefore, KK-262/C met the requirements for vaccine strains and was recommended for the manufacturing of vaccine preparations [21].

### 2.3. Seroimmunotype III

Studies with virulent ASFV strain Mozambique-78 were initiated in VNIIVViM in 1978. On the basis of its immunobiological characteristics, this strain was designated as a reference strain of seroimmunotype III. After selective passages of Mozambique-78 in the PBMC culture, attenuated hemadsorbing strain MK-200 was obtained [22].

In the PBMC culture, the MK-200 strain accumulated in titers of 10^6.5^–10^7.5^ HAU_50_/mL. It had moderate reactogenicity in pigs, and caused a rise in body temperature up to 40.2 °C in 20% of the pigs for 4–5 days after intramuscular administration at a dose of 10^6.0^–10^7.0^HAU_50_. On day 14, 90% of the pigs developed resistance to infection with the parental virulent strain of Mozambique-78 at a dose of 10^3.0^ HAU_50_.

Three lots of the 25× concentrated (PEG 6000) virus-containing cultural suspension were used to assess the harmlessness of strain MK-200. Three groups of 8 piglets (of 2–4 months of age) were inoculated intramuscularly with a virus dose of 10^8.5^–10^9.0^ HAU_50_. From days 3–5 after administration, 30% of the piglets had an increase in body temperature up to 40.5 °C without manifestation of other clinical signs of the disease.

The distribution dynamics of ASFV strain MK-200 in the organs of piglets was also investigated. On days 7 and 14 after administration, the virus was detected in the blood, lungs, liver, kidneys, spleen, lymph nodes, thyroid gland, and bone marrow in titers from 10^1.0^ to 10^4.0^ HAU_50_/mL (g). On days 21–30, the virus was detected only in samples of the blood, liver, and spleen in titers of 10^1.0^–10^1.5^ HAU_50_/g. On days 7–14, the minor hyperplasia of the bronchial lymph nodes and spleen was visually detected. At later dates post inoculation, these pathologic changes were not registered. Naïve pigs kept for two months in contact with pigs inoculated with MK-200 died after inoculation with virulent strain Mozambique-78. This fact indicated that the attenuated MK-200 strain is not transmissible from pig to pig. On the basis of these results, strain MK-200 was recommended as a vaccine strain of ASFV seroimmunotype III.

The relatively long viremia after inoculation of some of the attenuated ASFV strains, recommended as vaccine strains, caused serious concerns about their use in animals with weakened immunity and in pregnant sows. Detailed studies on sows from 70 to 96 days of gestation were carried out with the experimental series of the vaccine made using strain MK-200. The vaccine was administered at a dose of 10^8.5^ HAU_50_. On day 5 after inoculation, the body temperature of the sows reached 41.2–41.7 °C, and severe inhibition and food refusal were observed. In the saliva, urine, and feces samples taken from sows 6–11 days after vaccination, ASFV was isolated in titers of 10^1.0^–10^2.0^ HAU_50_/mL (g). One of the four sows farrowed on day 17 after vaccination and delivered 8 normally developed piglets. The ASFV titer in the sows’ blood was 10^4.0^ HAU_50_/mL; in the placenta, 10^3.5^ HAU_50_/mL; in fetal fluid, 10^2.5^ HAU_50_/mL. In the blood of newborn piglets, ASFV was detected in titers of 10^1.5^–10^2.5^ HAU_50_/mL. Subsequently, the ASFV was not detected in the blood, secretions, and excretions collected from piglets during the two-month observation period. Three other sows aborted 6–7 days after vaccination. ASFV accumulated in their blood in titers of 10^4.5^–10^6.5^ HAU_50_/mL, in the placenta with 10^3.5^ HAU_50_/mL, and in fetal fluids with 10^2.0^–10^2.5^ HAU_50_/mL. In samples of blood and parenchymal organs obtained from 26 aborted fetuses, ASFV was isolated in 18 cases in titers of 10^1.0^–10^2.0^ HAU_50_/mL (g) [13]. On the basis of these results, it is not recommended to use attenuated strain MK-200 for the vaccination of pregnant sows.

### 2.4. Seroimmunotype IV

The most extensive studies on the development of live vaccines against ASF were conducted with the virus of seroimmunotype IV. This was due to the fact that ASF epizootics in the 1960s–1980s in a number of European countries, in Latin America, and in the former USSR (Odessa region) were caused by viruses of seroimmunotype IV [23].

Attenuated strain FK-32/135 was selected by passaging of the virulent reference strain France-32 in PBMC culture [24]. The inoculation of FK-32/135 induced the protection of pigs against other virulent ASFV isolates of seroimmunotype IV: Cuba-71, Cuba-80, Malta-78, Sao-Tome and Principe-79, DNOPA-Luanda (from Angola), Brasil-80, and Odessa-77. Strain FK-32/135 was distinguishable from the majority of virulent and attenuated ASFV strains by pronounced “loose” hemadsorption in PBMC and LS cultures.

On day 7 after the intramuscular injection of strain FK-32/135 at a dose of 10^7.0^ HAU_50_, the virus was detected in the blood of pigs in titers of 10^1.0^–10^2.0^ HAU_50_/mL, and on day 14, the virus was not detected in the blood. Pathological and morphological changes in the lungs were manifested on days 4 and 10 by foci of catarrhal inflammation, followed by normalization of the lung structure by day 21.

The possibility of the formation of protection against ASFV in newborn piglets vaccinated within the first hours of life before their first colostrum feeding was investigated. Strain FK-32/135 (as a suspension of PBMC culture) was orally administered to piglets at a dose of 10^7.3^ HAU_50_, or injected intramuscularly at a dose of 10^6.7^ HAU_50_/mL. The clinical status of the newborn piglets after administration of the virus was within normal limits, and no lags in growth and development were observed. It was also demonstrated that 80%–100% of newborn piglets, obtained from sows inoculated with strain FK-32/135, were able to form a defense against ASFV of seroimmunotype IV after being vaccinated.

Various forms of experimental live vaccines based on strain FK-32/135 were studied: native, concentrated, lyophilized, and emulsified.

The 25× concentrated form of the FK-32/135 “culture vaccine” (prepared using PEG 6000), with a dose for intramuscular administration of 10^8.3^ HAU_50_, induced the protection of 80%–100% of the pigs by day 7 after vaccination, with a duration of at least four months. This vaccination did not affect the formation of protective immunity after subsequent vaccination against other viral diseases such as classical swine fever, Aujeszky’s disease, and swine vesicular disease.

To induce fast protection against ASFV, the pigs were intramuscularly inoculated with a highly concentrated (100×, by PEG 6000) vaccine preparation from strain FK-32/135 at a dose of 10^9.2^ HAU_50_. Pigs were intramuscularly challenged with virulent strain France-32 at a dose of 10^4.0^ HAU_50_ on day 1, 3, 5, or 7 after vaccination. Protection was formed in 100% of the pigs three days after vaccination.

Studies on aerosol vaccination were of particular interest. For this purpose, piglets of two months of age were placed in an isolated chamber, where the 25× concentrated (by PEG 6000) vaccine from FK-32/135 strain, with a titer of 10^8.5^ HAU_50_/mL, was then dispersed by an aerosol generator for 5–10 min. For the aerosol vaccination, the minimal dose of the vaccine that protects pigs from the control infection with virulent strain France-32 was 10^6.7^ HAU_50_. Protection in these pigs was formed on day 4 after vaccination and lasted for 120 days (observation period). The maximal accumulation of the virus in the pigs’ organs was observed on the second day after aerosol vaccination: lungs, 10^6.3^ HAU_50_/g; and bronchial and mediastinal lymph nodes, 10^3.0^–10^4.0^ HAU_50_/g. After 14 days and at later dates, no virus was isolated from the pigs’ organs and tissues.

During the first 6–9 days after aerosol vaccination, an increase of body temperature up to 41.2–41.5 °C was observed in 15% of the pigs, but the general condition of those pigs was satisfactory.

Along with the development of native cultural virus vaccines based on strain FK-32/135, studies were also conducted on the production of lyophilized preparations. Piglets of 2–3 months of age were aerosolized or intramuscularly injected with a lyophilized vaccine preparation from strain FK-32/135 at doses of 10^6.7^ and 10^7.0^ HAU_50_, respectively. In 2–4 days after aerosol vaccination, the virus was detected in the lungs, kidneys, heart, spleen, tonsils and bronchial, mediastinal, and submandibular lymph nodes. The maximal accumulation of the virus was found on day 2 after vaccination in the lungs, and bronchial and mediastinal lymph nodes in titers of 10^6.3^, 10^4.1^, and 10^3.0^ HAU_50_/g, respectively. On day 7, the virus was only detected in the spleen in a titer of 10^1.3^ HAU_50_/g. From day 10 to 4 months after aerosol vaccination, the virus could not be isolated.

In the first 2–4 days after intramuscular administration of the lyophilized vaccine, the virus was localized in the lungs, spleen, tonsils, and bronchial, mediastinal, portal, pharyngeal, and parotid lymph nodes. On day 4 after vaccination, a relatively high accumulation of the virus was detected in the lungs, bronchial lymph nodes, and spleen in titers of 10^3.4^, 10^2.9^, and 10^2.5^ HAU_50_/g, respectively. On day 7, the virus persisted only in the lungs, and bronchial and mediastinal lymph nodes, but in lower titers. Ten days after vaccination, the virus was isolated only from parotid lymph nodes. During a period from 14 days to 6 months after intramuscular vaccination with the lyophilized vaccine, no virus was isolated from the pigs’ organs and tissue.

Emulsified forms of the vaccine were developed for the possibility of long-term storage and subsequent operational use. The emulsified preparations were stable for at least five years.

### 2.5. Seroimmunotype V

The representative strain of seroimmunotype V TSP-80 was obtained by Dr. W. Plowright from Kiravira-67 isolate (Tanzania-67), isolated in Tanzania in 1967. Kiravira-67 demonstrated heteroimmunotypic properties and consisted of at least four ASFV seroimmunotypes, one of which is strain TSP-80 [25]. According to the results of HADIA and the immunobiological test (see Introduction), this strain was different from the reference strains of seroimmunotypes from I to IV, and caused the death of pigs on days 5–9 after intramuscular inoculation at a dose of 10^3.0^ HAU_50_. From strain TSP-80 by passaging in cell cultures of kidney cells of a pig (PPK-66b/17), CV, and PBMC, using various selection methods (limiting dilutions, selective sorption, selection by heat sensitivity, and virus passaging in the presence of pigs sera specific to serotypes I, III, and VI (active in HADIA)), attenuated strain TSP-80/300 (seroimmunotype V) was obtained [25]. This strain was weakly reactogenic and did not revert when inoculated to pigs in a mixture with strain LK-111 (seroimmunotype I). During five consecutive passages in pigs, it caused the development of protection against the homologous virulent ASFV on the 14th day after vaccination. The accumulation of the virus in the blood of vaccinated pigs on days 7–14 was 10^3.5^–10^5.0^ HAU_50_/mL. On day 30, the virus was not detected in the blood. The strain maintained its characteristics during 30 consecutive passages in the PBMC culture. Eight series of the concentrated (by PEG 6000) experimental vaccine were prepared using strain TSP-80/300. The vaccine was tested on 100 pigs from 15 days to 12 months of age. Results demonstrated that the experimental vaccine was harmless, low-reactogenic, and, by the 14th day after vaccination, induced the protection of pigs against a challenge with homologous virulent strain TSP-80 at a dose of 10^4.0^ HAU_50_ [26].

The main characteristics of live culture vaccines against the ASFV of seroimmunotypes I–V are presented in Table 2.

Studies on the development of live vaccines against ASFV isolates and strains from seroimmunotypes VI–VIII were limited to the characterization of the obtained attenuated strains.

### 2.6. Seroimmunotype VI

From the blood of a piglet resistant to ASFV of seroimmunotype III and subsequently infected with isolate Tanzania-67, the virulent strain TS-7 was isolated. TS-7 was designated as a reference strain of seroimmunotype VI because it was different from the strains of seroimmunotypes I–V [25]. Passaging TS-7 in cultures of PBMCs and PPK-66b led to the selection of attenuated hemadsorbing strain TS-7/230. By the 14th day after intramuscular administration of TS-7/230 at a dose of 10^7.0^ HAU_50_, all pigs were protected against a challenge with virulent ASFV strain TS-7.

### 2.7. Seroimmunotype VII

Virulent strain Uganda caused the death of 50% of pigs intramuscularly infected at a dose of 10^4.0^ HAU_50_. According to the results of HADIA and immunobiological tests, it was classified as a reference strain of seroimmunotype VII. The Uganda strain was passaged in PBMC and PPK-66b cultures with selection of variants with “loose” hemadsorption. At passage level 50, attenuated strain UK-50 was obtained. After intramuscular administration to pigs at a dose of 10^6.0^ HAU_50_, it caused a temperature reaction in only 9% of the inoculated pigs. On days 7–14 after vaccination, the titers of the virus in the blood of pigs were in the range of 10^1.5^–10^3.0^ HAU_50_/mL. After challenge on day 14 with virulent strain Uganda at a dose of 10^4.0^ HAU_50_, a temperature reaction was observed in only 33% of the pigs. On days 10–40, viremia was 10^1.5^–10^3.0^ HAU_50_/mL. The virus did not revert in the mixture with strain LK-111 (seroimmunotype I) during three consecutive passages in pigs.

### 2.8. Seroimmunotype VIII

According to the results of HADIA and immunobiological tests in pigs, the Rhodesia strain, obtained in 1984, was classified as a reference strain of ASFV of seroimmunotype VIII. Its passaging in PBMC and LS cultures led to the selection of attenuated variant RK-30. Pigs inoculated with RK-30 at a dose of 10^3.0^ HAU_50_ did not develop any clinical signs of the disease, but, two weeks after infection, viremia (up to 10^5.0^ HAU_50_/mL) was detected. By the 14th day after infection with RK-30, the pigs became resistant to infection by virulent strain Rhodesia at a dose of 10^3.0^ HAU_50_. Pigs twice intramuscularly inoculated with strain RK-30 at a dose of 10^6.5^–10^7.0^ HAU_50_ (administered at an interval of 14 days) were also protected against challenge with strain Stavropol-01/08 of seroimmunotype VIII, representing ASFV currently circulating in the Russian Federation [27].

## 3. Conclusions

Earlier, we saw that pigs could be protected from challenge with virulent viruses following infection with natural lowly virulent viruses, or with viruses attenuated by passage in tissue culture or by deletion of genes involved in virulence [4,6,7,28,29].

Among naturally attenuated ASFV strains, the most well-known and thoroughly studied are OURT88/3 and NH/P68 [4,7,29,30,31].

Avirulent strains Georgia07∆9GL&DP96R/UK, Ba71∆CD2/EP402R, Benin∆MGF, Benin∆DP148R, and NH/P68∆A238L were obtained by genetic modifications, and they provided protection against homologous and sometimes heterologous ASFV strains (according to the genotypic classification of the ASFV) [32,33,34,35].

Intensive research on the creation of live vaccines based on attenuated ASFV strains has been carried out in FRCVM (formerly VNIIVViM) for about twenty years. There is no universal method for the generation of attenuated strains, but the necessary prerequisite was multiple passaging of the original virulent reference strain in the PBMC culture and/or cultures of continuous cell lines [36]. The limiting dilutions, adsorption of the most strongly binding virions on sensitive cells, and removal of these virions from the viral population were used as selection methods. Virus subpopulations demonstrating the “loose” hemadsorption were selected for further passaging. As a result, we obtained attenuated strains of ASFV of seroimmunotypes I–VIII, protective preparations against ASFV of seroimmunotypes I–V, and reference sera specific to ASFV of serotypes I–IX, which were active in HADIA. The attenuated ASFV strains of seroimmunotypes I–V, selected as candidate vaccine strains, were not transmissible from inoculated to naïve pigs kept together, did not revert during three to five consecutive passages in pigs, and were weakly reactogenic.

Attenuated strains of ASFV can cause the development of a chronic form of the disease. This was frequently observed in pigs vaccinated with a Portuguese ASFV isolate weakened by sequential passages in PBMC cultures [11]. Of all pigs inoculated with a naturally attenuated ASFV isolate (ASFV/NH/P68), 25%–47% developed chronic lesions and a disease characterized by a delayed onset of fever, viremia, and high levels of antiviral antibodies with severe hypergammaglobulinemia [29]. Immunopathological conditions involving hypergammaglobulinemia and systemic immune activation were observed in pigs inoculated with other moderately virulent ASFV isolates [37,38]. Less pronounced clinical reactions, including fever and joint swelling, were described for ASFV strain OURT88/3 [7,39].

Intramuscular administration of attenuated strains of seroimmunotypes I–VIII to clinically healthy pigs did not cause chronic ASF. Viremia and clinical and pathomorphological manifestations of ASF were observed in a period not exceeding 14–30 days post inoculation.

Results of the massive use of live vaccines in the 1960s gave reason to believe that the immune status of pigs may affect the consequences of the introduction of attenuated ASFV strains. Vaccination of pregnant pigs with attenuated strain MK-200 led to the manifestation of clinical signs of ASF, abortions, and the death of most sows. Attenuated nonhemadsorbing variant Kc-160, usually avirulent for healthy pigs, caused the death of 20% of pigs with lowered immune status. These facts indicate that experimental live vaccines based on attenuated strains must undergo laboratory tests on pigs with different immune-system status. A method of modeling the immunodeficiency status of pigs is γ-irradiation with a dose of 4.0 gray, which leads to the development of acute radiation sickness of moderate severity. Inoculation of attenuated strain FK-32/135 to γ-irradiated pigs did not cause clinical signs of the disease and ensured their protection against subsequent infection with virulent strain France-32. On the other hand, inoculation of attenuated strain MK-200 caused the death of about 50% of immunodeficient pigs and provided only partial protection against their subsequent infection with a virulent strain of Mozambique-78 [40].

Differences between attenuated strains were also manifested in the timing and level of viremia, reactogenicity, and pathological and morphological changes in vaccinated pigs. Among all of the attenuated strains obtained at FICVM (formerly VNIIVViM), FK-32/135 demonstrated the best characteristics.

In the process of research, the inoculation of attenuated strains and challenge at a 100% lethal dose, as a rule, was carried out intramuscularly. This was necessary to reliably control the doses of the administered viruses. In a number of studies, methods were used that were more similar to natural conditions: oral or contact infection. In future studies, we should investigate all of the above methods of vaccination and challenge.

At present, the prospects of using live vaccines against ASF are not clearly defined because, along with the positive aspects, they have a number of significant drawbacks: virus shedding, development of postvaccination complications, and, in some cases, insufficient protection of immunocompromised animals. The infection of vaccinated pigs with a virulent ASFV strain of a homologous seroimmunotype sometimes led to its “engraftment” and virus carrying.

The predominant opinion is that live vaccines are unlikely to ever be in demand since pig farms are discrete, and the timely slaughter of livestock in the outbreak site, in combination with compliance with veterinary and sanitary measures, could reliably interrupt the spread of the virus. The use of live vaccines in wild boars in regions where ASF is not an endemic disease can exacerbate the problem of disease control.

Nevertheless, the accumulated knowledge of the immunobiological properties of ASFV, and a unique collection of virulent and attenuated strains of the eight seroimmunotypes, opens up new perspectives for further research using molecular biology methods to develop protective preparations for limited use [41].

Currently, due to the identification of genes for protectively significant ASFV proteins, the targeted creation of live vaccines against ASFV with programmable properties is becoming a reality [7,42].

## Figures and Tables

**Table 1:** Seroimmunotype classification of African swine fever virus (ASFV) strains, isolates, and variants.

Group	Reference Strain	ASFV Strains, Isolates, and Variants	
		Highly Virulent and Moderately Virulent	Low Virulent and Avirulent
**Seroimmunotype I**			
**1**	**Lisbon-57**	Lisbon-57 (L-57), Kimakia, Katanga-78, Katanga-105, Katanga-115, Madeira-65, Diamant	LS, L-50, LF-97, Kimakia-155, Diamant-160, Katanga-139 (Kc-139), Katanga-149 (Kc-149), Katanga-160 (Kc-160), LK-111, Katanga-350
**Seroimmunotype II**			
**2**	**Congo-49**	Congo-49 (K-49), Yamba-74, Le Bry-73, Sylva	NVL-1, Mfuati-79, Ndjassi-77, KK-202, KK-262/C
**Seroimmunotype III**			
**3**	**Mozambique-78**	Mozambique-78 (M-78), MK-101	MK-200, MK-210
**Seroimmunotype IV**			
**4**	**France-32**	France-32 (F-32), Cuba-71, Brazil-80, Cuba-80, Malta-78, Sao-Tome and Principe-79 (STP-1), DNOPA-Luanda, Odessa-77	FK-32/135
**Seroimmunotype V**			
**5**	**TSP-80** (obtained from ASFV isolate Kiravira-67)	TSP-80	TSP- 80/300
**Seroimmunotype VI**			
**6**	**TS-7** (obtained from ASFV isolate Kiravira-67)	TS-7	TS-7/150 TS-7/230
**Seroimmunotype VII**			
**7**	**Uganda**	Uganda	UK-50, UK-80
**Seroimmunotype VIII**			
**8**	**Rhodesia,** **Stavropol 01/08**	Rhodesia, Stavropol 01/08	RK-30, RK-UF, St-CV_1_/20
**Seroimmunotype IX**			
**9**	**Davis**	Davis	None
**Isolates of which the serotypic affiliation, according to hemadsorption inhibition assay (HADIA) data, does not correspond to immunobiological test results**			
**10**	**None**	Portugal-60 (P-60), Spain-70 (according to data of HADIA belonging to Serotype IV)	P-60/801
**Heterogeneous isolate**			
**11**	**Kiravira-67**	Kiravira-67	None
**Isolates that were not studied, and untyped**			
**12**	**None**	Bartlett, Nanyuki	None

**Table 2:** Characteristics of live culture vaccines against ASFV of seroimmunotypes I–V.

Seroimmunotype	Reference Strain	Vaccine Strain	Protection Percentage*/ Term (Days after Vaccination)	Viremia (Day 14 after Immunization), HAU_50_/mL
I	**Lisbon-57**	Katanga-350	50/7 80/14–180	10^0.7^
II	**Congo-49**	KK-262/C	80–100/14–180	10^2.0^–10^3.5^
III	**Mozambique-78**	MK-200	50/7 82–92/14–150	10^3.5^–10^4.0^
IV	**France-32**	FK-32/135	80–100/10–180	No
V	**TSP-80**	TSP-80/300	80–100/14–120	10^3.5^–10^4.5^

Note: * Control intramuscular infection with homologous virulent reference strain at a dose of 10^3.0^–10^4.0^ HAU_50_.
